# Gene sequencing and *in silico* characterization of mannose-binding lectin in South African chicken breeds

**DOI:** 10.5455/javar.2025.l955

**Published:** 2025-09-22

**Authors:** Peter Ayodeji Idowu, Takalani J. Mpofu, Oliver T. Zishiri, Ogun Joshua Oluwamayowa, Khathutshelo A. Nephawe, Bohani Mtileni

**Affiliations:** 1 Department of Animal Sciences, Faculty of Science, Tshwane University of Technology, Pretoria, South Africa; 2 Department of Genetics, Westville Campus, University of KwaZulu-Natal, Durban, South Africa; 3 Institute of Animal Breeding and Husbandry, University of Kiel, Kiel, Germany

**Keywords:** Indigenous chicken, innate immunity, genetic diversity, ligand binding, MBL gene sequencing

## Abstract

**Objective:**

To sequence and characterize the mannose-binding lectin gene of three South African chicken breeds, namely, Potchefstroom Koekoek (PK), Venda (VN), and Ovambo (OV), to ascertain their genetic and immunologic diversity.

**Materials and Methods:**

Total RNA was isolated from hepatic samples, quantified, and reverse-transcribed to generate cDNA. The *MBL* gene was amplified by PCR, confirmed by gel electrophoresis, purified, and sequenced using Sanger sequencing. Sequences were analyzed with FinchTV and submitted to GenBank. Comparative sequences were retrieved from National Centre for Biotechnology Information for multiple sequence alignment and phylogenetic analysis using MEGA. ProtParam and ExPASy were used for physicochemical analysis. Secondary structures were predicted using PDBsum, while tertiary structures were modeled with Swiss-Model, refined by GalaxyWEB, and validated by ProSA. Functional domain analysis, binding site prediction, and ligand interaction studies were also performed.

**Results:**

MBL sequences showed breed-specific differences in protein length, isoelectric points, and thermostability. PK and VN MBLs had acidic *pI* values (< 7), while OV displayed a higher, alkaline *pI*. Conserved Glu–Pro–Asn (EPN) and Trp–Asn–Asp (WND) residues, linked by calcium ions, were identified for mannose-binding. Phylogenetic analysis revealed that PK breeds clustered closely with the White Leghorn (95%), OV clustered nearby PK breeds and Leghorn (82%), while VN clustered more closely with Indian Assel breeds (96%). Two conserved motifs (IPR033990 and IPR001304) were detected. Secondary and tertiary structures revealed predominant random coils in PK and OV, and more alpha-helices in VN. Binding site analysis identified key regions likely involved in immune modulation.

**Conclusion:**

This research reveals variation in *MBL* genes and their immune relevance in South African chicken breeds, offering a basis for breeding strategies.

## Introduction

South African chicken breeds are known to have unique characteristics such as resistance to disease and preservation of genetic resources traits suited to local conditions [1]. Among these breeds, Potchefstroom Koekoek (PK), Ovambo (OV), and Venda (VN) are the most predominant breeds used by poultry farmers in South Africa due to their adaptability, resilience, and significance in smallholder farming systems [2,3].

Despite their widespread usage, little or no molecular study has been done on their immunological traits, such as the mannose-binding lectin (*MBL*) protein. Gaining insights into the genetic variability, structural characteristics, and functional properties of *MBL* in these breeds is important for advancing both scientific knowledge and breeding strategies. Most especially given their exposure to diverse pathogens under free-range and low-input production systems [1,3]. Therefore, characterizing their *MBL* gene could provide insights into the diversity and capacity of South African chicken breeds’ innate immune system.

Generally, chickens are known to consist of animal lectins [4,5]. These lectins contribute to antimicrobial defense, cell communication, and pathogen recognition [6,7]. Based on their carbohydrate recognition domains (CRDs), animal lectins can be categorized into three distinct types. This includes p-type, I-type, and c-type [8]. The p-type is found mainly in tissues with a preference to bind to the galactose or N-acetyl galactosamine region of pathogens and is the main factor involved in cell adherence, development, and immune defense [9]. The I-type lectin (galectin) binds primarily to beta-galactoside sugars such as lactose and N-acetyl lactosamine, with a major role in cancer progression, tissue development, and wound healing [9]. C-type lectins, mainly expressed in liver cells, preferentially bind carbohydrates like fucose, mannose, and N-acetylglucosamine. Mannose-binding lectin (*MBL*), a soluble Ca^2^⁺-dependent member of this family, plays a key role in pathogen recognition, immune regulation, and clearance of apoptotic cells [10–12]. It specifically binds terminal mannose and other carbohydrate-rich residues and is found on the surface of pathogens like Gram-positive and Gram-negative bacteria and, in some cases, parasites, viruses, yeast, parasites, and mycobacteria [12,13]. Upon binding to these sugar residues, *MBL* neutralizes pathogens by marking them for immune recognition and further initiates the lectin pathway of the complement system [10,14]. This process leads to opsonization and enhanced phagocytosis, which ultimately results in the clearance of the pathogens from the host cell [4,15]. Finally, *MBL* can differentiate between self-cells, non-self-cells, and apoptotic cells to ensure targeted immune responses without harming host tissues [10,14,15].

Bodi et al. [16] reported that *MBL* exists as a trimeric structure, having a molecular weight of approximately 96 kilodaltons, composed of three identical 32 kilo Dalton subunits. Each subunit contains a collagen-like domain, an N-terminal nitrogen-rich cross-linking segment, and a C-terminal CRD [9,10,15]. These domains assemble into a classical triple-helical structure [10]. In chickens, *MBL* is capable of forming multiple oligomeric states, ranging from dimers to hexamers [15,17], with the homotrimer identified as the fundamental building block [18]. The trimer consists of three identical polypeptide chains, forming a collagen-like triple helix with globular lectin domains at the C-terminal end [19]. By binding a calcium ion, each lectin domain can specifically interact with sugars like N-acetyl-D-glucosamine, mannose, N-acetyl-mannosamine, fucose, and glucose [20].

Each chicken *MBL* (c*MBL*) subunit is organized into several structural regions: a collagen-like region, a neck domain, a CRD, and a linker region [9,10,21]. The collagen-like region, characterized by Gly-XY repeats, is critical for trimer assembly and interaction with *MBL*-associated serine proteases to induce complement pathway activation [20,22,23]. Connecting the collagen-like domain and the CRD, the neck region contributes to the stability of the trimer [10,15], while the CRD contains a conserved carbohydrate-binding site for sugar recognition [24]. Oligomerization of *MBL* increases the number of CRDs, enhancing multivalent ligand binding [25]. Also, most *MBL* variants possess a linker region between the neck and CRD, providing flexibility for optimal carbohydrate binding [26].

Chicken *MBL* contains an EPN (Glu-Pro-Asn) motif in its CRD, enabling binding to D-mannose, L-fucose, and GlcNAc [12,27]. Ligand-binding motifs are highly diverse across species; for example, saltwater clam (*Glycymeris yessoensis*) lectins exhibit motifs such as Glu–Pro–Asp (EPD), Gln–Pro–Gly (QPG), Gln–Pro–Ser (QPS), Tyr–Pro–Gly (YPG), and Tyr -Pro -Thr (YPT) [28]. *MBL* also interacts with other immune molecules like dendritic cells, pentraxins, and the serum amyloid p component, reinforcing its role in bridging innate and adaptive immunity [29,30].

Therefore, this study aimed to sequence and characterize the *MBL* gene in selected South African indigenous chicken breeds using gene sequencing and *in silico* approaches. Through computational analyses, the study explored the physicochemical properties, subcellular localization, functional domains, evolutionary relationships, secondary and tertiary protein structures, and potential ligand-binding sites of the cMBL protein. The objective was to gain insights into the immunological diversity of MBL and its role in breed-specific indigenous South African chicken breeds. By promoting sustainable approaches to poultry farming and reducing antibiotic dependence, these findings not only enhance poultry health and productivity but also contribute to achieving the UN Sustainable Development Goals of zero hunger and good health and well-being.

## Materials and Methods

### Ethical approval

The study was approved by the Animal Research Ethics Committee of the Faculty of Science, Tshwane University of Technology (AREC2021/10/002; date: October 18, 2021), and conducted in accordance with ARRIVE guidelines and local animal welfare regulations.

### Animal tissue and total RNA extraction

Liver samples from three South African chicken breeds were collected and stored at –80°C in liquid nitrogen. Total RNA was extracted, quantified, and quality-checked before synthesizing cDNA using a first-strand synthesis kit. qPCR was conducted using two primer sets targeting fragments of 544 bp (5’-GAT AAG CCG GAA AAC CCT GAA-3’ / 5’-GTT ACA ACA ATT CCA CGT TCT CCT-3’) [31] and 835 bp (5’-GGT AAA GGT GCT GAT CTG TGG-3’ / 5’-TGA GAG AAG AAA GTT GGA TTT-3’) [32].

### PCR amplification and sequencing

The *MBL* gene was sequenced following PCR amplification, product purification, Sanger sequencing, and sequence analysis. Genomic DNA was extracted and amplified using NEB OneTaq 2X Master Mix with Standard Buffer (New England Biolabs, M0482S). Each 20 µl PCR reaction contained 10 µl of Master Mix, 1 µl of genomic DNA (20 µg/µl), 1 µl each of forward and reverse primers (10 µM), and 7 µl of nuclease-free water (E476). Thermal cycling included an initial denaturation at 94°C for 5 min, 35 cycles of 94°C for 30 sec, 50°C for 30 sec, 68°C for 60 sec, and a final extension at 68°C for 10 min, followed by storage at 4°C until further use.

### Gel electrophoresis and visualization of PCR products

PCR product integrity and size were evaluated on a 1% agarose gel stained with EZ-Vision^®^ Bluelight DNA Dye and visualized using a gel documentation system to confirm successful amplification.

### PCR product purification using the ExoSAP method

PCR products were purified enzymatically using the ExoSAP method. For each reaction, 10 µl of amplified DNA was combined with 2.5 µl of an ExoSAP mixture containing Exonuclease I (20 U/µl; NEB M0293L) and Shrimp Alkaline Phosphatase (1 U/µl; NEB M0371). The mixture was incubated at 37°C for 15 min to remove residual primers and dephosphorylate unused nucleotides, followed by enzyme inactivation at 80°C for 15 min.

### Sanger sequencing reaction and post-sequencing cleanup

Purified PCR fragments were sequenced using the BrilliantDye^™^ Terminator Cycle Sequencing Kit v3.1 (Nimagen, BRD3-100/1000) following the manufacturer’s protocol. Sequencing products were then cleaned using the ZR-96 DNA Sequencing Clean-up Kit (Zymo Research, D4053) to eliminate unincorporated dye terminators and salts.

### Capillary electrophoresis and sequence data analysis

Sequencing analysis was performed on an Applied Biosystems ABI 3500XL Genetic Analyzer equipped with a 50 cm capillary array and POP-7 polymer. The resulting chromatograms were visualized and interpreted using FinchTV, a freely available software designed for high-quality electropherogram viewing. To support comparative analysis, the corresponding coding sequences of the *MBL* gene were retrieved from other chicken *MBL* sequences available in the National Centre for Biotechnology Information (NCBI) Protein Database (https://www.ncbi.nlm.nih.gov/protein) [33]. The derived sequence data from this study have been submitted to GenBank and are available with the following accession numbers: PP782170 (PK), PP782171 (OV), and PP782172 (VN).

### Sequence analysis

The Expasy server (https://web.expasy.org/translate) [34] was used to convert the nucleotide sequences to amino acid sequences (proteins), and the longest open reading frame (ORF), which is highlighted in red, was selected for this study. This ORF with the highest red is known to contain the full protein-coding sequence, with the longest length and position, making it suitable for further protein analysis [34].

### Prediction of amino acid sequences and functional characterization

Amino acid sequences were inferred from nucleotide sequences using the ExPASy Translate tool. The resulting protein sequences were analyzed with ExPASy Protein tools to determine their properties, including consensus motifs for chicken *MBL*. Functional domains were mapped, and the protein’s ontology and classification were predicted using the InterPro server, which catalogs homologous protein domain families (https://www.ebi.ac.uk/interpro/) [35,36].

### Multiple sequence alignment and phylogenetic analysis

For evolutionary analysis, seven *Gallus gallus* chicken mannose-binding lectins *cMBL* with one *MBL* from a plant as an outgroup were retrieved from the NCBI with accession numbers AF231714.1, KF469209.1, KU378610.1, KU378616.1, KF469210.1, KF469208.1, JF717877.1, and KC329532.1 (https://blast.ncbi.nlm.nih.gov/Blast.cgi?PAGE=Proteins), with three generated protein sequences from this study, PP782170 (PK), PP782171 (OV) and PP782172 (VN), making a total of ten [10] c*MBL*. Phylogenetic trees were produced in MEGA software 11.0.21 using the maximum likelihood test with a bootstrap test of 1,000 replicates [37].

### Physicochemical property analysis

The ProtParam tool (http://web.expasy.org/protparam/[34]) was used to determine physicochemical characteristics of *MBL* from South African chicken breeds, including molecular weight (MW), aliphatic index (AI), grand average of hydropathy (GRAVY), isoelectric point (*pI*), and conserved signal peptide sites (SPCS).

### Subcellular location and solubility prediction

The probable subcellular localization of the protein was assessed using CELLO (http://cello.life.nctu.edu.tw/[38]). Protein solubility and hydrophobic regions were evaluated with SOSUI (http://harrier.nagahama-i-bio.ac.jp/sosui/ [39]), where hydrophobic segments were annotated as potential transmembrane regions. Signal peptide cleavage sites were predicted using the TOPCONS server (http://topcons.cbr.su.se/pred [40]).

### Predictions and validations of the secondary and tertiary structures of proteins

To gain insights into protein function, the secondary structures were predicted using the PDBsum database (https://www.ebi.ac.uk/thornton-srv/databases/pdbsum/) [41], while tertiary structures were modeled using the SwissModel tool for homology study (https://swissmodel.expasy.org/) [42]. These models were further refined with the GalaxyWEB refiner tool [43], which uses ab initio methods to refine loop and terminal regions. The predicted structures were validated with PDBsum, which identified the amino acid sequences involved in forming secondary structures, such as α-helices, β-sheets, coils, and loops. This provides insights into both structural and functional aspects of *MBL*. Lastly, ProSA (Protein Structure Analysis) was used to predict potential errors in the 3D models provided in PDB format [43,44].

### Prediction of binding sites

The Galaxysite tool [45] was employed to predict possible ligand-binding sites within the protein’s tertiary structure. This tool does not only predict the most likely binding pockets but also suggests potential ligand molecules that may interact with these sites. These predicted ligand interactions provide valuable insights for future therapeutic research and functional characterization of the *MBL* studies.

## Results

### Primary structure and subcellular location of selected South African chicken breeds

Table 1 shows the MW, which was 27, 27 and 33 kDa, and the SPCS were 29–30, 28–29 and 29–30 for the PK, VN and OV breeds. The number of amino acids was 251, 254 and 313 and the aliphatic index observed was 77.73, 79.49 and 79.74 for the PK, VN and OV breeds. The instability index observed were 30.88, 29.57 and 24.18 and the GRAVY of the protein sequences predicted were  −0.520, −0.468 and −0.415 for PK, VN and OV breeds. This study further observed that both the PK and VN breeds are located in the periplasmic subcellular with an isoelectric *pI* of 5.85. In contrast, the OV breed is located in the cytoplasmic inner membrane, with a *pI* of 8.90.

### Multiple sequence alignment of cMBL of selected South African chicken breeds

**Figure 1.**
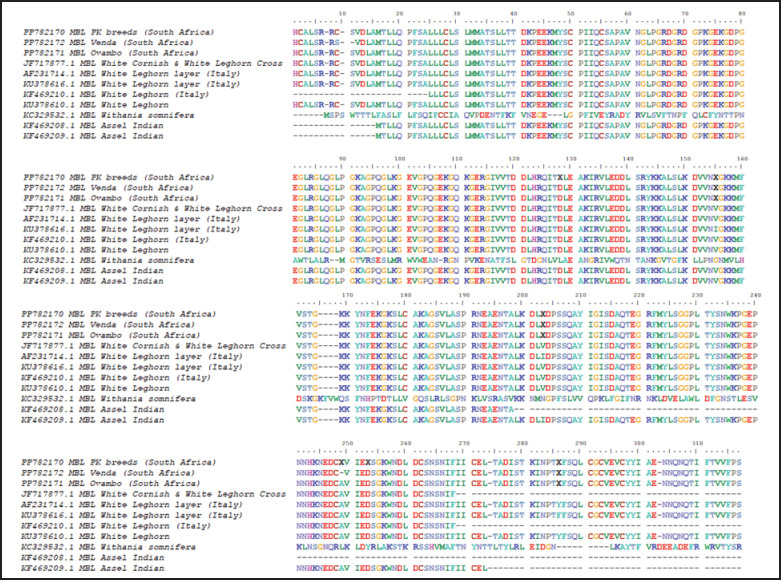
Alignment of mannose-binding lectin protein sequences from chicken breeds. Sequences were aligned using MEGA 11 by Clustal W; Residues that are identical or share similar properties are highlighted using consistent color.

Figure 1 shows the multiple sequence alignment of the *MBL* gene. The contoured area shows the site of the signal peptide. All the chicken breeds sampled in this study have gaps at the 10th, 165th to 168th, and 274th positions. Also, PK, VN, and OV have 6, 3, and 3 insertions in all 317 protein sequences with respect to other chicken breeds. This shows that these three breeds are closely related to each other. In the three breeds studied, the MBL protein sequences were highly conserved.

### Prediction of the functional domain of the South African cMBL

**Figure 2.**
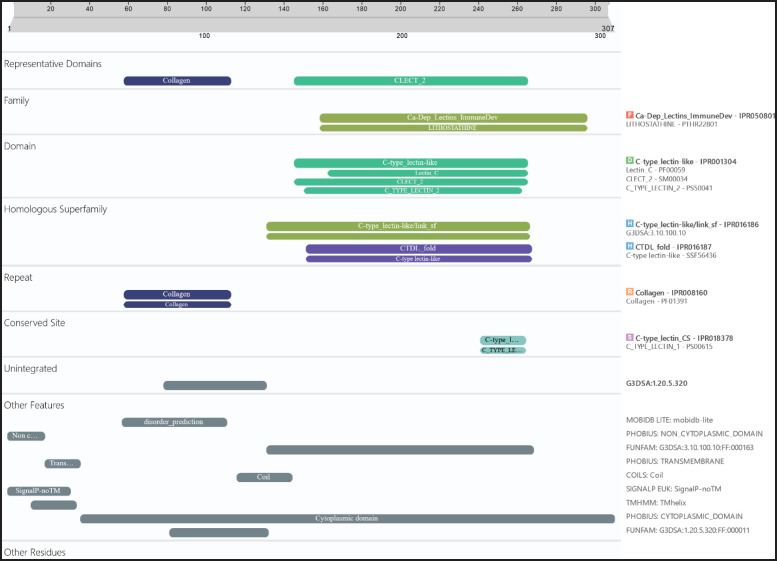
Prediction of the functional domain of South African chicken mannose-binding lectin observed by the InterPro server.

Two functional domains were identified using the InterPro server (https://www.ebi.ac.uk/interpro/), IPR033990 and IPR001304, in the amino acid sequence of the chicken *MBL* (Fig. 2). The two functional domains, IPR033990 (collectin domain), also called c-type lectin domain (CTLD), and the second, IPR001304 (c-type lectin domain), were observed in all the chicken breeds sampled. The representative domains are collagen and C-LECT-2 and the domain observed in this study is c-type lectin-like.

**Table 1.** Analysis of the primary structure and physicochemical properties of MBL from South African chicken breeds using the ProtParam server.

Chicken Breeds	SPCS	No aa	MW	*pI*	GRAVY	Instability Index	Aliphatic -Index
PK	29–30	251	27092.81	5.85	−0.520	30.88	77.73
VN	28–29	254	27362.16	5.85	−0.468	29.57	79.49
OV	29–30	313	33572.29	8.90	−0.415	24.18	79.74

SPCS: Signal Peptide-Conserved Sites, No. aa: Number of amino acids, GRAVY: Grand Average of Hydropathicity: *pI*: Isoelectric point. MW: Molecular weight. PK=Potchefstroom Koekoek breed, VN= Venda breed, and OV=Ovambo breeds. Chromosome number: 6

### Phylogenetic analysis of the MBL protein sequence of South African chicken breeds

To investigate the relationships and variation among c*MBL* genes, a phylogenetic tree was generated using MEGA 11. The maximum likelihood method was applied to infer evolutionary relationships. The computed data indicated that there are nine different clusters, including the outgroup. South African breeds PK and Ovambo clustered closely with European lines (White Leghorn and White Cornish White Leghorn Crossbred). This suggested shared genetic ancestry despite geographic separation. The Indian breeds Assel and White Leghorn layer formed a distinct regional cluster, suggesting localized evolutionary lines. The VN breed from South Africa occupied a separate cluster, highlighting significant divergence from other African and European breeds. This may reflect unique selection pressures or genetic isolation. The plant species *Withania somnifera* *MBL* was included as a reference for rooting the phylogenetic tree.

### Predictions and validations of the secondary and tertiary structures of proteins

The PROCHECK tool in PDBsum was used to evaluate the predicted tertiary structures of MBL proteins from three chicken breeds (Table 2, Fig. 1). The PK breed cMBL contains 15.5% β-strands, 27.9% α-helices, and 56.6% other structural elements. The OV breed has a similar composition with 15.4% β-strands, 28.3% α-helices, and 56.3% remaining components. The VN breed differs slightly, comprising 17.6% β-strands, 20.8% α-helices, a short helix of 1.3%, and 60.4% other elements.

Based on the amino acid sequence of the protein, PROCHECK predicts its secondary structure and compares it with the secondary structure shown in the crystallographic data. The secondary structure elements that are projected to occur are often beta strands, loops, and alpha helices. The procheck result shows the overall quality of the protein structure (Fig. 4).

**Table 2.** Prediction of secondary structure and possible compositions of the three indigenous chicken breeds from South Africa.

Breeds	Strand	Alpha helix	Others	Total residues
PK	39 (15.5%)	70 (27.9%)	142 (56.6%)	251
OV	39 (15.4%)	72 (28.3%)	143 (56.3%)	254
VN	55 (17.6%)	65 (20.8%)	193 (60.4% + 1.3% Helix)	331

PK: Potchefstroom Koekoek; OV: Ovambo; VN: Venda.

The stereochemical quality of the predicted MBL protein structures was assessed using Ramachandran plot analysis, which showed that 92.6%, 93.7%, and 95.3% of residues fell within the most favored regions for the PK, OV, and VN breeds, respectively. Additionally, ProSA, a web-based tool for detecting errors in protein 3D structures, was employed. The proteins yielded *z*-scores of −5.91, −6.36, and −6.16, indicating high-quality models (Fig. 5). The high proportion of residues in favored regions confirms strong agreement between predicted and observed secondary structures, and the *z*-scores indicate the models are reliable and acceptable [46].

### Prediction of binding sites

The galaxy site prediction tool revealed possible binding sites and potential interacting ligands (Table 3). Figures 6, 7, and 8 show the possible noncovalent interactions (such as hydrogen bonds, salt bridges, and hydrophobic bonds) for the predicted ligands of the MBL proteins.

**Figure 3.**
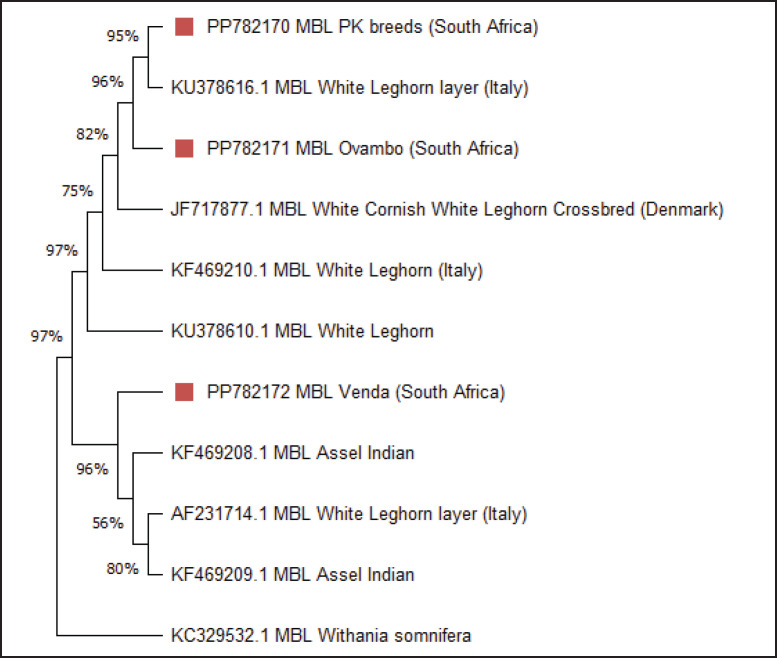
Maximum-likelihood phylogenetic tree based on protein sequences of chicken (*Gallus gallus*) from various breeds, with 1000 bootstrap replicates. The protein sequences of different breeds were retrieved from the NCBI database, alongside three breeds from this study with accession numbers PP782170 (PK), PP782171 (OV), and PP782172 (VN). *MBL* *Withania somnifera* was used as the reference/outgroup.

## Discussion

The isoelectric *pI* of a protein reflects the pH at which it carries no net electrical charge [47]. In this study, the MBL protein of the OV breed had an alkaline *pI* (8.90), while the PK and VN breeds had acidic *pI* (5.85). This variation indicates differences in their net charge under physiological conditions [48]. These differences suggest that the OV MBL may be less mature or structurally distinct compared to the PK and VN MBL. Also, OV and *pI* are negatively charged in acidic environments and positively charged in alkaline environments, while PK and VN are positively charged in acidic environments and negatively charged in alkaline environments [49]. Previous study has reported that most premature proteins tend toward alkalinity, whereas mature proteins generally exhibit more acidic *pI* distributions [50]. This could be attributed to post-translational modifications or differences in amino acid composition [51]. The relatively higher *pI* of the OV MBL protein indicates a lower proportion of acidic residues (aspartic acid and glutamic acid) or a higher content of basic residues (lysine and arginine) [52]. In contrast, the lower *pI* values in PK and VN suggest these proteins are more negatively charged [48]. These physicochemical differences may influence how the MBL proteins interact with pathogens, ligands, or immune components across the chicken breeds.

**Figure 4.**
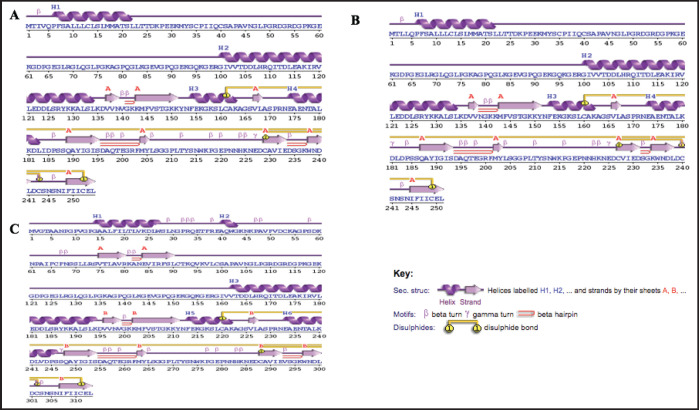
The PROCHECK tool from PDBsum predicted the secondary structures of the proteins. PK (A), OV (B), and VN (C).

**Figure 5.**
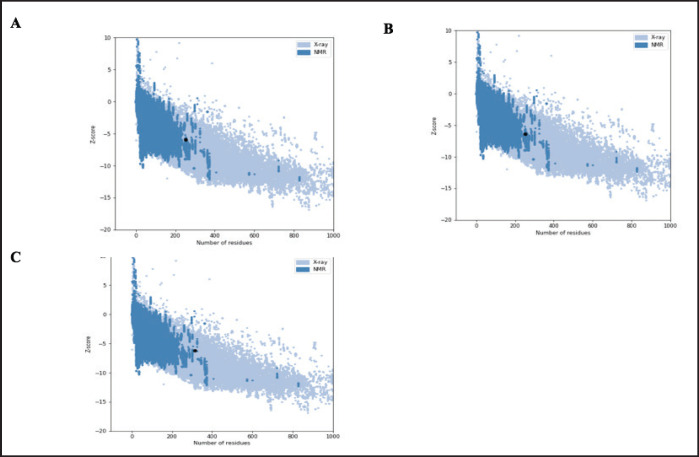
ProSA plot with different *z* scores of PK (A), OV (B), and VN (C). The black dot represents the position of the protein structure in comparison with the standard parameters for proteins of an equivalent size.

A significant correlation has been observed between a protein’s isoelectric point (*pI*) and its subcellular localization [50]. The acidic *pI* values of PK and VN MBLs align with their predicted localization in inner membrane compartments [53]. This environment typically favors hydrophilic and acidic proteins, which support solubility and interactions with other biomolecules [54]. On the other hand, the alkaline *pI* of the OV MBL protein suggests greater hydrophobicity, which potentially facilitates stronger interactions with membrane lipids [55] and enhances signaling stability and structural anchoring [53,56]. These variations are likely driven by breed-specific evolutionary changes, such as amino acid substitutions, insertions, or deletions within the *MBL* gene [33].

**Table 3.** Binding sites and potential ligands of the MBL protein predicted by the Galaxy tool for the three chicken breeds.

Ligand Name	Binding sites
Alpha-D-glucopyranose (GLC)	187Q 215K 218E 222H 225E 236N 237D 238L
2-acetamido-2-deoxy-beta-D-galactopyranose (NGA)	187Q 215K 218E 220N 222H 225E 236N 238L
alpha-L-fucopyranose (FUC)	218E 222H 225E 236N 237D 238L

The MW of the cMBL protein observed in the present study for the PK, VN, and OV were 27, 27 and 33.5 kDa, respectively. These values are close to the range of the theoretical MW, which ranges from 25 to 27 kDa for processed MBL using native mass spectrometry [57]. Also, OV MW is very close to the MW of 32 kDa reported by Ulrich-Lynge et al. [12]. The study of Zhang et al. [57] also reported that the molecular weight of Ross Broiler chicken breeds was 26 kDa. The differences observed in South African chicken breeds may suggest breed-specific modifications. In other species, the molecular weights of human MBL, pumpkin [*Cucurbita pepo *MBL and wild garlic (*Allium ursinum*)] MBL were 31 kDa, 22.6* kDa,* and 14.83 kDa, respectively [33,58,59]. Variations in the MW of the MBL protein in this study could be attributed to species specificity and glycosylation [54]. Nevertheless, differences in the MW of MBL in comparison to other chicken breeds sampled could reflect the underlying genetic diversity that influences immune system functionality and overall health [4,16]. Also, this variation could be a result of a single-nucleotide polymorphism, which affects the protein’s structure and function [60,61]. In detail, variations in the promoter region and exon 1 of the *MBL* gene cause differences in *MBL* oligomerization, which subsequently impact the molecular weight of the protein. It is important to know that the higher the molecular weight, the greater the degree of polymerization [61]. This will, in turn, influence the functional capacity of *MBL* in pathogen recognition and complement system activation [62].

**Figure 6.**
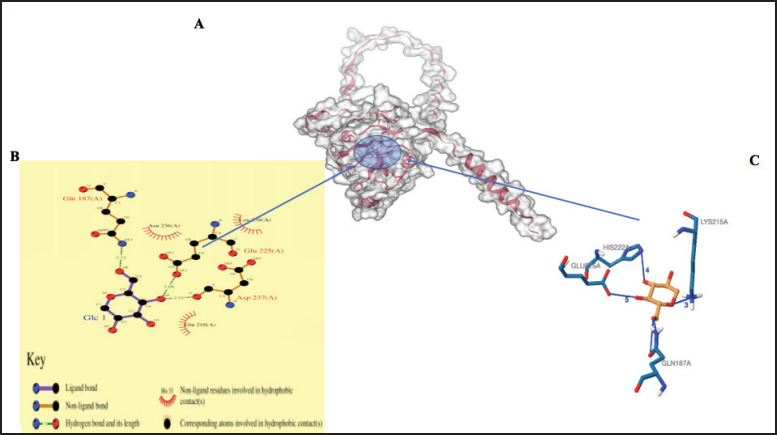
Three-dimensional structure of MBL protein predicted by Swiss Model. The interaction within the binding site with Alpha-D-glucopyranose (GLC) is predicted by the galaxy prediction tool (A). The ligand interaction plots (B). The interaction chains predicted by the Protein Interaction Ligand Profile tool (C).

**Figure 7.**
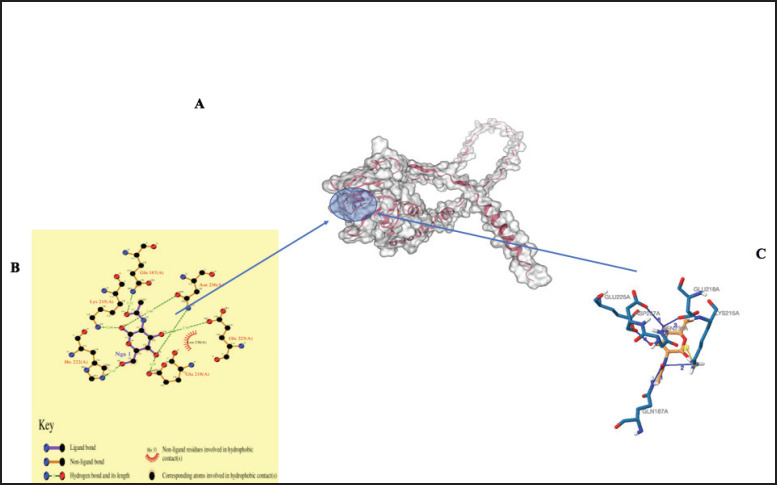
Three-dimensional structure of MBL protein predicted by Swiss Model. The interaction within the binding site with 2-acetamido-2-deoxy-beta-D-galactopyranose (NGA) is predicted by the galaxy prediction tool (A). The ligand interaction plots (B). The interaction chains predicted by the Protein Interaction Ligand Profile tool (C).

The phylogenetic analysis of the *MBL* gene in South African chicken breeds depicts some level of variation of the *MBL* gene. The PK and Ovambo breeds show close genetic affinity with White Leghorn and White Cornish crossbreds (European lines). This clustering could reflect historical gene flow or a similar *MBL* gene introduced during breeding programs [37]. Such genetic conservation suggests that these South African indigenous breeds could possess immune characteristics related to the commercial chicken breeds. In contrast, the VN breed exhibits clear genetic separation from PK and OV, clustering instead with Indian native breeds (Assel). This divergence may reflect unique local adaptations, limited introgression from commercial breeds, and the preservation of ancestral genetic signatures [63]. Also, adaptation to similar environmental situations and the influence of uncontrolled mating systems in indigenous chicken populations may have influenced the c*MBL* gene variation observed in VN [30]. Consequently, each protein could have evolved due to differences in breed response to diseases, thus leading to antigenic variation [64]. These findings align with broader studies of South African indigenous chickens, which report moderate to high genetic differentiation among local lines and emphasize the presence of multiple maternal lineages, including those tracing back to the Indian subcontinent [65]. This study suggests that OV and VN breeds are genetically distant from each other, explaining the diversity within South Africa’s chicken genetic resources.

The functional domain called collectin domain IPR033990 (CTLD) was found at positions 141-251, 142-253, and 201-313 in the MBL protein sequence of PK, VN and OV chicken breeds. Similar collectins are mostly found in human collectin, lung surfactant protein, liver collectin, and MBL [59]. They can bind carbohydrates on the surface of the pathogen, necrotic or apoptotic cells, and allergens [10,59]. Also, they mediate activities such as phagocytosis [61], identifying the high-rich mannose region and triggering the killing of cells [59].

**Figure 8.**
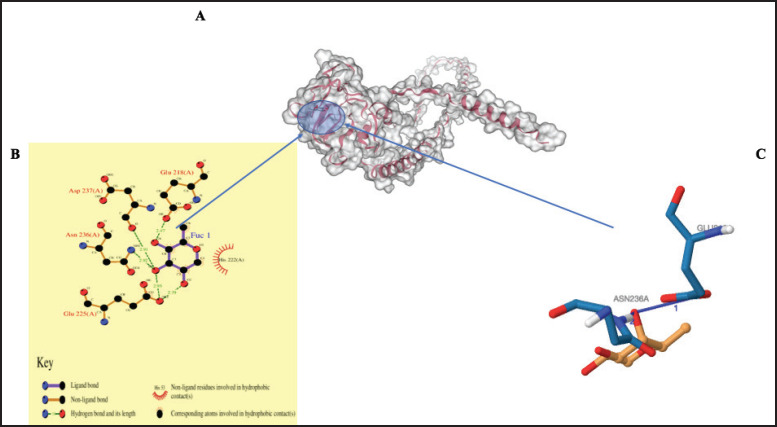
Three-dimensional structure of MBL protein predicted by Swiss Model. The interaction within the binding site with alpha-L-fucopyranose (FUC) is predicted by the galaxy prediction tool (A). The ligand interaction plots (B). The interaction chains predicted by the Protein Interaction Ligand Profile tool (C).

The functional domain IPR001304 (c-type lectin) was found in the regions 133–250, 132–253 and 191–312 in the cMBL protein sequence of the PK, OV and VN chicken breeds, respectively. The c-type lectin is first characterized in some animal lectins, known to be a Ca^2^⁺-dependent recognition domain, also known as the CTLD, consisting of about 110–130 residues. It consists of four perfectly conserved cysteines and two disulfide bonds. Both domains have two representative domains, namely collagen and C-LECT-2. The conserved regions in several animal lectins mostly consist of a Ca^2^⁺-dependent carbohydrate recognition domain that is shared by several distinct protein families [6,8,10]. The two functional domains are later divided into subdomains. The collectin domain has both c-lect and collectin-like, while the c-type has lectin 2, lectin c, and c-lect 2. The homologous family is the CTDL fold IPR016187 and the lectin-like c-type IPR016186. The MBL is among the few c-type lectin families that have the c-type lectin domain and the conserved domain of Ca^2^⁺, which binds to the sugar or mannose region [25].

The CRD observed in the present study shows that the cMBL protein contains all the sequence characteristics of C-type lectin with great homology with other cMBL from a previous study [58]. The four cysteines (Cys) that maintain the distinctive double-loop structure are the two most significant structural components of C-type lectins [19,66]. The c-type lectins consist of two structural elements. The first is the four cysteines with the aim of stabilizing the double-loop nature (Cys170-Cys184, Cys193-Cys213) and the 161-EPN-163, 171-WND-181 motifs essential for ligand binding under the supervision of a calcium ion present in the binding pocket region of MBL, which is important for the specificity of MBL [67]. This finding aligns with several studies [5,27] regarding the cysteine region present in cMBL under the influence of Ca^2^⁺-dependent binding affinity.

In this study, the protein secondary structure was categorized into four types: α-helix, extended strand, β-turn, and random coil. For the PK breed, α-helices were the most prevalent, followed by random coils, extended strands, and β-turns. In contrast, for the OV and VN breeds, random coils were dominant, with α-helices, extended strands, and β-turns occurring in decreasing order.

The impact of the electrostatic bulkiness of the R group could result in coil formation [67]. Jimenez et al. [33] observed similar phases in a study on MBL structure in *Allium* species.

Understanding protein function requires accurate prediction of both secondary and tertiary structures, which is essential for identifying functional sites and protein–ligand interactions. In this study, homology modeling was used due to its effectiveness in predicting three-dimensional protein structures from available templates [68]. Model quality was assessed using the *z*-score, which reflects the overall structural reliability and conformational energy. All three cMBL protein models exhibited negative *z*-scores, with their positions in the blue region of the plot, indicating high-quality structural models [69]. Regarding the protein model, the structural component of a protein is directly related to its function and the internal underlying force, depending on its interaction with other molecules [70]. Identifying binding sites on proteins is essential in understanding their function, interactions, and potential roles in various biological processes [71]. Glycosidic bonding is of two types: [1] the N-linkage, which occurs when the asparagine side chain attaches nitrogen atoms to the sugar surface, and [2] the O-linkage, which occurs when the serine side chain attaches oxygen atoms to the sugar surface. The MBL region proposed that Asn236, Leu225, Glu218, Gln187, and Asp237, with the ligand name Glc, are the major residues involved in the binding of the mannose region. The major proteins that occupy the glycosylation region that are involved in binding are the Glutamic acid Glu187, Asp237, and Gln187. Also, there is a projection that Glu218, Asp236, Gln187, His222, Lys215, and Leu236, with the ligand name Nga, are the residues observed to be involved in the binding of the mannose region to the MBL region and occupy the glycosylation region except for Leu236. Lastly, in the MBL region, Asp237, Asn236, Glu225, and His222 with ligand name Fuc1 were the main residues involved in carbohydrate binding and are the proteins found in the glycosylation region, except for His222. The Asn and Asp have been reported to be important residual proteins for carbohydrate recognition [62]. Therefore, three ligand names were predicted as the site where mannose binds to lectin (glycosylation) in this study. These ligand areas were also observed in a previous study [27,28].

## Conclusion

This study successfully combined gene sequencing and *in silico* characterization to investigate the *MBL* gene in three indigenous South African chicken breeds. Using computational tools. The physicochemical properties, functional domains, subcellular localization, secondary and tertiary structures, and evolutionary relationships of the cMBL protein were analyzed. The results revealed breed-specific structural features and conserved functional motifs, revealing the immunological significance of MBL in these local breeds. Understanding the genetic and structural diversity of *MBL* contributes valuable insights into breed-specific traits with implications for improving poultry health and productivity. Furthermore, this study supports sustainable livestock development goals by highlighting the potential of indigenous breeds in enhancing disease resilience without over-reliance on antibiotics. To understand the interaction between the protein and sugar region (mannose), protein modeling will be recommended for future study to elucidate the immunomodulatory roles of MBL across different chicken populations.

## List of abbreviations

AI, aliphatic index; AREC, Animal Research Ethics Committee; ARRIVE, Animal Research: Reporting of In Vivo Experiments; cMBL, chicken mannose-binding lectin; CRD, carbohydrate recognition domain; CTLD, C-type lectin domain; GlcNAc, N-acetyl-D-glucosamine; GRAVY, grand average of hydropathicity; ExPASy, Expert Protein Analysis System; IPR, InterPro protein domain/family identifier; kDA, kilo Dalton; MASP, MBL-associated serine proteases; MBL, mannose-binding lectin; MEGA, Molecular Evolutionary Genetics Analysis software; MW, molecular weight; NCBI, National Center for Biotechnology Information; ORF, open reading frame; OV, Ovambo; PDB, Protein Data Bank; *pI*, isoelectric point; PK, Potchefstroom Koekoek; ProSA, Protein Structure Analysis; SAP, serum amyloid P component; SPCS, signal peptide-conserved sites; VN, Venda.
